# Bleaching Teeth with Peroxides of Hydrogen and Sodium

**Published:** 1902-05-15

**Authors:** Joseph Head

**Affiliations:** Philadelphia


### BLEACHING TEETH WITH PEROXIDES OF HYDROGEN AND SODIUM.

BY JOSEPH HEAD, M.D., D.D.S., PHILADELPHIA.

Peroxid of Hydrogen and peroxid of sodium when heated give off oxygen in great quantities. This nascent oxygen at the moment, when it leaves the compound, is most caustic, and, therefore, if we can liberate this gas directly on a tooth, we shall be able to remove any organic stain. Either pyrozone, which is a 25 percent solution of peroxid of hydrogen, or sodium dioxide can be used. These two materials seem equally powerful, but they are somewhat different in their actions, and, therefore, would better be described separately.

If the stained tooth is pulpless and the apical ends of the canals have been tightly sealed, the treatment should be as follows: Apply the rubber dam and tie one if not two ligatures around the neck, so that leakage is impossible. Let the rubber dam go slightly over the nostrils of the patient to prevent the fumes of the nascent oxogen from irritating the air passages. Oil should be rubbed on the hands of the operator and on the face of the patient. The tooth should be dried internally and externally. Cotton soaked in pyrozone should be packed in the canal and a hot ball burnisher, such as is used in plastic work, should be placed against the cotton so that the steam of the nascent oxygen will be driven through the tooth substance. After the cotton becomes dry, it should be removed, and the tooth heated again internally with hot air, when the process described above can be repeated several times. Finally, cotton soaked in pyrozone should be sealed in the canal with hard gutta percha, so that any gas that is given off may by pressure be driven throughout the dental tubules.

Then we are ready for the second stage of the process. The enamel should be thoroughly dried with hot air blasts and heated instruments until the patient feels the heat of the tooth in the gum. This makes the oxygen within the tooth canal exert great pressure; then a piece of cotton soaked in pyrozone should be placed on the enamel, and a broad, hot instrument held against it, so that the steam shall be driven in from the outside. This should be continued until the cotton becomes dry, when the enamel should be ironed with a highly heated ball burnisher. This drives out the pyrozone that the enamel has soaked up, and in driving it out, liberates the nascent oxygen within the enamel substance. The effect of this last-mentioned ironing is most marked and the stains can be seen perceptibly to whiten. This process can be repeated in its various stages as often as desirable, and when the patient leaves, fresh pyrozone should be sealed in the canal with gutta percha, in order that the bleaching process may continue until the next visit. If, finally there is a slight stain that the nascent oxygen will not remove, a strong solution of oxalic acid should be used in the same way. The oxalic acid is not only a powerful oxidizer of organic material, but will also change any iron stain to a colorless oxalate.

What has been said about the use of pyrozone, with a few cautions, applies equally well to peroxid of sodium, as both bleach primarily by setting free nascent oxygen. The peroxid of sodium is most valuable where oil in the tooth is to be saponified, and, therefore, it will sometimes succeed where pyrozone fails; however, we must remember that when peroxid of sodium touches the soft tissues, it makes a deep burn, which pyrozone would not, and, therefore, great care should always be used that the sodium dioxide does not escape from the protection of the rubber dam. When this compound is placed in hot water, the oxygen is given off so rapidly that a distinct puff is heard, but when it is mixed on ice, a thick paste of the undischarged sodium dioxide can be obtained which when placed upon the dried enamel, and heated with a hot instrument will safely give off a tremendous quantity of nascent oxygen. This oxygen, as previously stated, will bleach the enamel of a tooth where the pulp is alive. When peroxid of sodium has been used it should be carefully washed off with water and neutralized with a weak acid. Before the rubber dam is removed, always be sure that none is left to burn the mouth. It is also for the same reason, dangerous to seal it up in a tooth canal unless the utmost precautions are taken against its escape.

Dr. Miller has, I believe, noted that concentrated peroxid of hydrogen under certain conditions, may attack the organic matrix that holds the enamel rods together.—This is a warning that should be noted and considered. But in spite of this warning, the good results obtained by me might be explained, even though Dr. Miller's observations were generally true.

Enamel that is sterilized of its infecting bacteria is better able to preserve its integrity by the use of antiseptic washes, even though the matrix may have been slightly weakened by the bleaching agent, than infected enamel, the matrix of which is being constantly thinned by bacteria that lie too deep for an ordinary wash to reach.

But whether this method even theoretically injures the enamel, is a subject for future research. This much, however, is sure, this method has permanently bleached stained enamel and that enamel has kept its normal color and integrity for over two years.—*Items of Interest.*

